# Production performance in cultivated mixed-sown grasslands combining *Poa pratensis* L. and various Poaceae forage grasses

**DOI:** 10.1371/journal.pone.0324084

**Published:** 2025-05-19

**Authors:** Sida Li, Wenhui Liu, Zhenghai Shi, Kaiqiang Liu, Wenhu Wang, Lingling Liu, Huimin Duan

**Affiliations:** 1 Key Laboratory of Superior Forage Germplasm in the Qinghai‐Tibetan plateau, Qinghai Academy of Animal Science and Veterinary Medicine, Qinghai University, Xining, China; 2 Laboratory for Research and Utilization of Qinghai Tibet Plateau Germplasm Resources, Xining, Qinghai, China; Chinese Academy of Forestry, CHINA

## Abstract

Kentucky bluegrass (*Poa pratensis* L.), a native grass species of the Qinghai-Tibetan Plateau, is widely used for ecological restoration due to its high growth rate and strong adaptability. However, monocultures of *Poa pratensis* are prone to rapid degradation and low productivity, limiting their suitability for animal husbandry. To address these challenges, this study evaluated the production performance and interspecific relationships of different mixed-sown and monoculture grasslands to identify optimal cultivation strategies. Field experiments were conducted over a six-year period, with three mixed-sown treatments—*Poa pratensis* combined with Siberian wildrye (*Elymus sibiricus* L.), Chinese fescue (*Festuca sinensis* Engler ex S.L.Lu), and alkali grass (*Puccinellia tenuiflora* (Griseb.) Scribn. & Merr.)—alongside their respective monocultures. LASSO regression (Least Absolute Shrinkage and Selection Operator Regression) and ROC curve analysis (Receiver Operating Characteristic Curve Analysis) were applied to identify key factors influencing production performance. The results indicated that the mixed-sown grassland of *Elymus sibiricus* and *Poa pratensis* significantly boosted forage yield by 216.88% to 323.06% in comparison with monoculture *Poa pratensis*. Additionally, the comprehensive evaluation index, which integrates forage yield and nutritional quality, was 16.41% higher for the *Elymus sibiricus* and *Poa pratensis* mixture than for the monoculture *Poa pratensis* grassland. These findings imply that the mixed-sown grassland of *Elymus sibiricus* and *Poa pratensis* effectively addresses the low productivity issue often seen in monoculture *Poa pratensis* grasslands. However, in terms of yield stability and interspecific compatibility, the mixed-sown grassland of *Puccinellia tenuiflora* and *Poa pratensis* demonstrated superior performance. Its relative total yield (RTY) consistently exceeded 1.0 from the third to the sixth year, reflecting higher interspecific compatibility and stable productivity over time. And the *Poa pratensis* and *Puccinellia tenuiflora* mixture showed the best performance, achieving the highest stability value of 3.12. Therefore, the combination of *Poa pratensis* and *Puccinellia tenuiflora* is recommended as the optimal strategy for achieving long-term yield stability and high productivity in cultivated grasslands.

## Introduction

The Qinghai-Tibetan Plateau, covering over 2.5 million km^2^ and averaging an elevation of more than 4000 m, is often referred to as the “Roof of the World” or the “Third Pole.” It plays a critical role in global and regional climate, hydrology, and ecosystems [1,2]. However, climate change, combined with the plateau’s harsh environment and infertile soils, has led to a decline in grassland forage production. This has pressured herders to increase livestock numbers to compensate for low meat production efficiency, exacerbating overgrazing and grassland degradation on the Qinghai-Tibetan Plateau [3,4]. Establishing perennial cultivated grasslands has emerged as an effective solution to counteract these challenges [5–7].

Kentucky bluegrass (*Poa pratensis* L.), a rhizomatous gramineous species, is widely distributed across the Qinghai-Tibetan Plateau. It possesses characteristics such as high growth rate, strong adaptability, and excellent wind resistance. Numerous studies have demonstrated that *Poa pratensis* can rapidly cover soil surfaces, prevent soil erosion, improve soil structure, and enhance soil carbon sequestration, as well as water and nutrient retention [8]. These attributes makes it an ideal species for ecological restoration on the Qinghai-Tibetan Plateau. Currently, *Poa pratensis* is widely used for restoring severely degraded grasslands, reclaiming mine sites, and supplementary sowing in lightly degraded grasslands on the plateau [9]. However, monoculture *Poa pratensis* grasslands suffer from rapid degradation and low productivity, limiting their application in animal husbandry [10]. Consequently, enhancing the productivity of *Poa pratensis* while maintaining ecological sustainability has become a key issue in addressing the forage shortage on the Qinghai-Tibetan Plateau.

Mixed grasslands present a more effective planting strategy compared to monocultures. Mixed grasslands involve the simultaneous cultivation of two or more crops forage species in the same land [11]. Recent studies have shown that mixed grasslands can significantly improve forage production and quality in high-altitude regions [12]. However, most current research focuses on legume-grass mixtures, where legumes enhance soil nitrogen content through biological nitrogen fixation, facilitating nutrient transfer to co-planted grasses and thereby increasing forage yield [13,14]. Despite these benefits, the high altitude, low accumulated temperature, and arid climate of the Qinghai-Tibetan Plateau pose challenges for many palatable perennial legumes, leading to slow regeneration rates and accelerated degradation of legume-based cultivated grasslands.

Therefore, the establishment of mixed grasslands comprising both perennial legumes and grasses may not be the optimal choice for the Qinghai-Tibetan Plateau. Instead, studies have suggested that mixed sowing of different gramineous forages can improve interspecific relationships among grasses [9]. This is achieved by strategically adjusting grassland composition to promote the coordinated development of individuals and populations, ultimately enhancing the efficiency of light interception and soil nutrients utilization. Furthermore, mixed sowing has been found to improve the stability and adaptability of soil microbial communities, enhance soil enzyme activity, promote nutrient cycling, and increase soil fertility [15].

In light of these findings, this study aims to explore the feasibility of establishing mixed grasslands dominated by different gramineous species through field experiments. This experiment utilizes gramineous forages widely distributed on the Qinghai-Tibetan Plateau with excellent adaptability, nutritional value, and forage yield. These include Siberian wildrye (*Elymus sibiricus* L.), Chinese fescue (*Festuca sinensis*), alkali grass (*Puccinellia tenuiflora*), and Kentucky bluegrass, to establish mixed grasslands. The objective is to utilize the characteristics of different forages to establish mixed grasslands with strong stability and high productivity, thereby achieving a win-win situation of protecting ecological functions and enhancing grassland productivity.

## 2. Materials and methods

### 2.1. Experimental site

The experiment was conducted at the National Perennial Herbage Germplasm Garden in Xihai Town, Qinhai Province, China (100°52.848′ E, 36°59.36′ N, 3156 m a.s.l.). This region experiences an average of 2980 hours of sunshine annually and has a mean annual temperature of 0.5 °C. Annual temperature fluctuations are significant, ranging from -27.3 °C to 25 °C.The mean annual precipitation is 369.1 mm, primarily concentrated in July, August, and September while the annual evaporation rate reaches 1400 mm. Before the experiment, the topsoil layer (0–20 cm) exhibited the following characteristics: pH 8.43, organic matter content of 32.48 g kg^-1^, total nitrogen content of 1.56 g kg^-1^, total phosphorus content of 1.39 g kg^-1^, available nitrogen of 88.8 mg kg^-1^, and available phosphorus of 2.2 mg kg^-1^. The natural grassland surrounding the experimental site is dominated by *Kobresia* species such as *Kobresia humilis*, *Kobresia pygmaea*, and *Kobresia capillifolia*, with associated vegetation including *Carex* spp., grasses (e.g., *Elymus nutans*), and forbs (e.g., *Polygonum viviparum*). The soil is characterized as a loamy chernozem.

### 2.2. Experimental design

The experiment included seven treatments: four monoculture grasslands—Siberian wildrye (*Elymus sibiricus* L.), Chinese fescue (*Festuca sinensis*), alkali grass (*Puccinellia tenuiflora*), and Kentucky bluegrass (*Poa pratensis* L.)—and three mixed-sown grasslands comprising *Poa pratensis* with *Elymus sibiricus* (*Poa + Elymus*), *Poa pratensis* with *Puccinellia tenuiflora* (*Poa + Puccinellia*), and *Poa pratensis* with *Festuca sinensis* mixture grassland (*Poa + Festuca*). All forage species used in this experiment, including *Elymus sibiricus*, *Festuca sinensis*, and *Puccinellia tenuiflora*, are native to the Qinghai-Tibetan Plateau and are classified as perennial grasses.

Sowing was conducted on July 15, 2018. Each plot covered an area of 50 m^2^ and was replicated three times. The total seeding rate for all treatments was 22.5 kg·hm^-2^. For the mixed-sown treatments, seeds were sown in a 1:1 weight ratio, with each species in the mixture receiving 11.25 kg·hm^-2^. The sowing process was carried out manually using a furrow drilling method with a row spacing of 30 cm. Before sowing, fertilizers were applied at rates of 75 kg·hm^-2^ of urea and 150 kg·hm^-2^ of diammonium phosphate. Weeding was conducted once in both the first and second years following seedlings emerged. The experiment was conducted under dryland conditions.

### 2.3. Sample collection and determination

From 2019 to 2023 (the 2nd to the 6th year), forage yield was assessed annually in September, when all forage species had reached the milk-ripe stage. In each experimental plot, five 1 m × 1 m quadrats with uniform growth were randomly selected and harvested at ground level. The harvested plant material from each quadrat was sorted by species in the field, and the fresh weight of each species was recorded. Subsequently, a 500 g fresh sample of each species was randomly selected. A portion of the sample was dried at 105°C for 30 minutes to determine moisture content, while the remaining portion was used to separately measure the fresh weight of stem and leaf components.

Acid Detergent Fiber (ADF, %) and Neutral Detergent Fiber (NDF, %) were measured using the Van Soest method (Van Soest et al., 1991). Crude Protein (CP) was determined by the Kjeldahl method (De Souza Nascimento et al., 2024), and Crude Fiber (CF) was analyzed using the gravimetric method (Van Soest et al., 1991).

Relative feed value (RFV) was calculated for each grass species based on the formula [10,15]:


Digestibledrymatter(DDM)=88.90−0.779×ADF(%)

$$Drymatterintake(DMI)=\frac{120}{NDF(\;\%)}$$

$$Relativefeedvalue(RFV)=\frac{DDM\times DMI}{1.29}$$

Where ADF (%) represents the acid detergent fiber content of the forage and NDF (%) represents the neutral detergent fiber content of the forage.

Relative total yield, relative yield, and interspecific competition intensity were determined simultaneously with the forage yield measurements [16].


$$Relativetotalyield(RTY):RYT=\frac{Y_{AB}}{Y_{AA}}+\frac{Y_{BA}}{Y_{BB}}$$

$$Relativeyield(RY):{RY}_{A}=\frac{Y_{AB}}{Z_{AB}\times Y_{AA}},{RY}_{B}=\frac{Y_{BA}}{Z_{BA}\times Y_{BB}}$$

$$Competitionratio(CR):{CR}_{A}=\frac{{(Y}_{AB}/Z_{AB})\times Y_{AA}}{{(Y}_{BA}/Z_{BA})\times Y_{BB}}$$

Where $Y_{AB}$ represents the biomass of species A in the mixture, $Y_{BA}$ represents the biomass of species B in the mixture, $Y_{AA}$ represents the biomass of species A in monoculture, $Y_{BB}$ represents the biomass of species B in monoculture, $Z_{AB}$ represents the proportion of species A in the mixture, and $Z_{BA}$ represents the proportion of species B in the mixture.

### 2.4. Statistical analysis

Data analysis was performed using SPSS 25.0 statistical software (SPSS Inc., Chicago, IL, USA) for ANOVA. Microsoft Excel 2019 (Microsoft Corp., USA) was used for data processing and statistical analyses. Mean separation was conducted using Tukey’s HSD test at a 5% significance level. Data visualization was carried out using Origin 2023 (Origin Lab Corp., USA). LASSO regression (Least Absolute Shrinkage and Selection Operator Regression) and the CRITIC weighting method (Criteria Importance Through Intercriteria Correlation) were applied using the web-based data science algorithm platform SPSSAU to determine the weights of the indicators. The comprehensive evaluation index was subsequently calculated using the following formula.


$$D=\sum_{i}^{k}\left(\frac{u_{ik}-X_{\min}}{X_{\max}-X_{\min}}\times \lambda_{k}\right)$$ (1)

Where $u_{ik}$ represents the measured value of the *k*-th indicator for the *i*-th treatment, $X_{\max}$ and $X_{\min}$ represent the maximum and minimum values of the *k*-th indicator across all treatments, and $\lambda_{ik}$ represents the weight of the *k*-th indicator.

## 3. Results

### 3.1. Comparative analysis of forage yield and stability in cultivated grasslands

**Fig 1:**
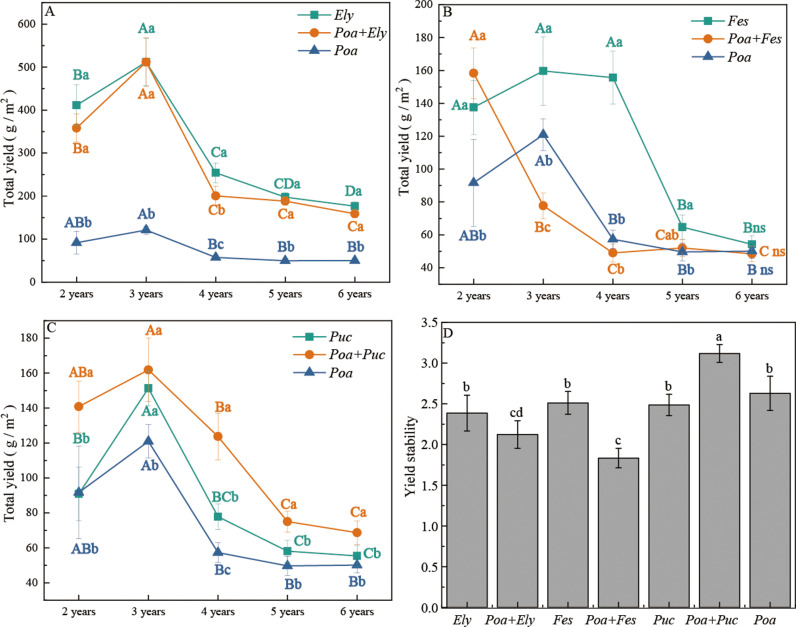
Forage yield of different cultivated grasslands. Note: Different letters in the figure indicate significant differences in forage yield among grasslands across different planting years (*P* < 0.05). (A) illustrates the forage yield of *Poa + Elymus* mixtures, *Poa* monoculture, and *Elymus* monoculture. (B) shows the forage yield of *Poa + Festuca* mixtures, *Poa* monoculture, and *Festuca* monoculture. (C) presents the forage yield of *Poa + Puccinellia* mixtures, *Poa* monoculture, and *Puccinellia* monoculture. (D) depicts the yield stability of different grasslands.

Different treatments had a significant effect on forage yield (Fig 1; *P* < 0.05). Forage yield in cultivated grasslands initially increased but later declined over time. No significant changes were observed from the 5th to the 6th year (*P* > 0.05). In the 4th year, the forage yield of the *Poa + Elymus* mixture was 21.33% lower than that of the *Elymus* monoculture (Fig 1A). From the 3rd to the 5th year, the forage yield of the *Poa + Festuca* mixture was significantly lower than that of the *Festuca* monoculture by 51.02%, 68.05%, and 19.44%, respectively (Fig 1B). In the 2nd, 4th, 5th, and 6th years, the *Poa + Puccinellia* mixture yielded 19.38%, 20.89%, 29.14%, and 23.97% more than the *Puccinellia* monoculture, respectively (Fig 1C). Overall, the *Poa + Elymus* mixture produced the highest total forage yield from the 2nd to the 6th year, whereas the *Poa + Festuca* mixture had the lowest yield.

Regarding yield stability, the *Poa + Puccinellia* mixture exhibited the highest stability value (3.12), with yield stability significantly increasing by 25.38% and 18.63% compared to the *Puccinellia* and *Poa* monocultures, respectively (*P* < 0.05). In contrast, the *Poa + Festuca* mixture had the lowest stability value (1.83), with yield stability significantly decreasing by 26.95% and 30.21% compared to the *Festuca* and *Poa* monocultures, respectively (*P* < 0.05) (Fig 1D, S1 Table in S1 File).

**Fig 2:**
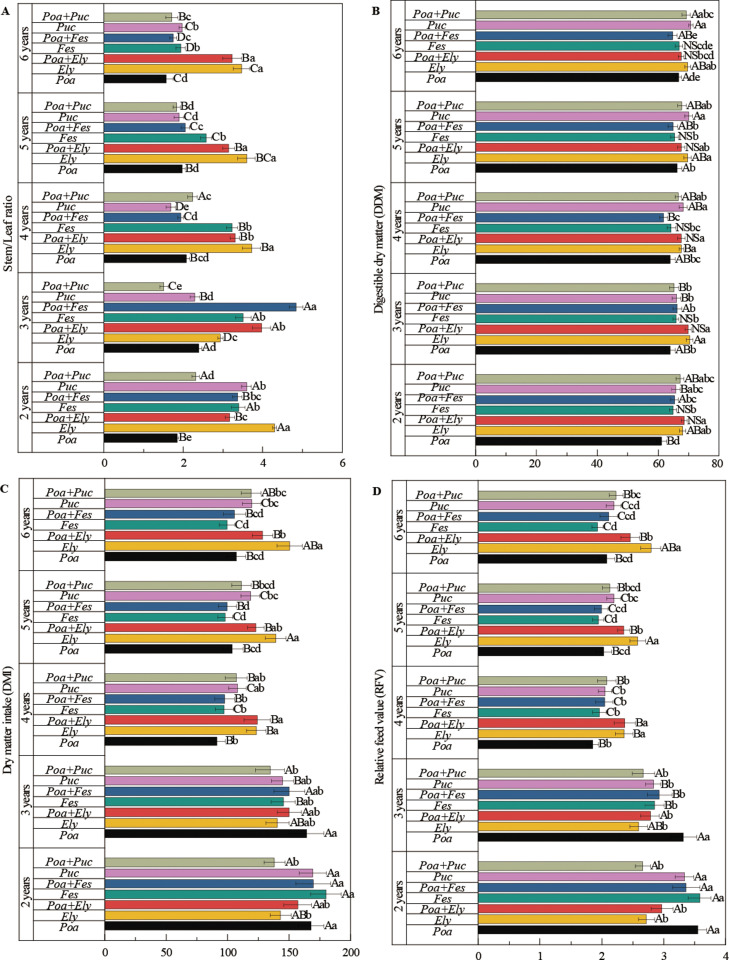
Stem/leaf ratio, digestible dry matter, dry matter intake and relative feed value of different cultivated grasslands. Note: Different uppercase letters indicate significant differences among different years within the same cultivated grassland (*P* < 0.05). Different lowercase letters indicate significant differences among different cultivated grasslands within the same year (*P* < 0.05).

**Fig 3:**
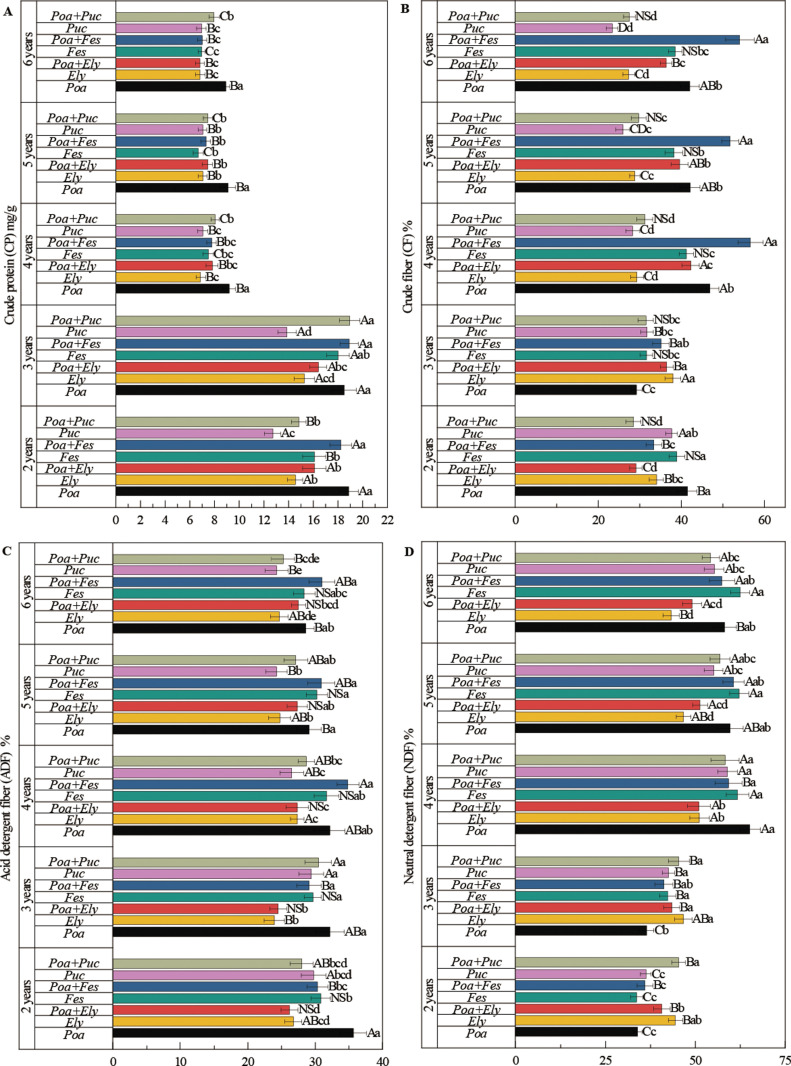
Crude protein, crude fiber, acid detergent fiber and neutral detergent fiber of different cultivated grasslands. Note: Different uppercase letters indicate significant differences among different years within the same cultivated grassland (*P* < 0.05). Different lowercase letters indicate significant differences among different cultivated grasslands within the same year (*P* < 0.05).

Treatment, year, and their interactions had a significant impact on biomass allocation and the nutritional quality of cultivated grasslands (Figs 2, 3). With prolonged cultivation, dry matter intake (DMI), relative feed value (RFV), and crude protein content gradually decreased (Figs 2C, 2D, 3A). In contrast, crude fiber, acid detergent fiber (ADF), and neutral detergent fiber (NDF) exhibited a consistent upward trend over time (Fig 3D, S2 Table in S1 File).

### 3.2. Interspecific interactions in mixed-sown grasslands with *Poa pratensis* L. and various Poaceae forages

**Fig 4:**
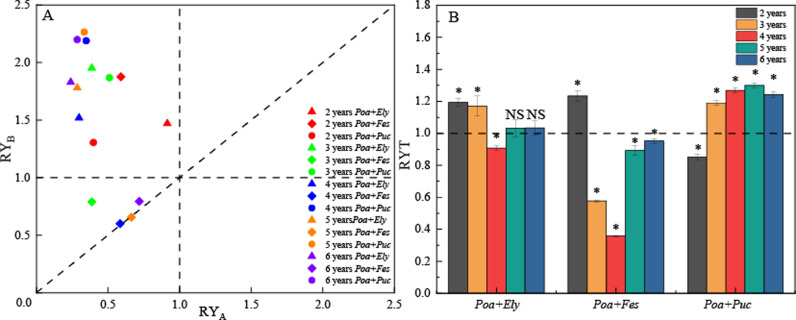
Interspecific relationships in different mixed grasslands. Note: (A) illustrates the relative yields of different mixed cultivated grasslands. (B) depicts the relative total yields of different mixed cultivated grasslands. “*” indicates a significant difference in RYT from 1 (P < 0.05).

In the mixed grasslands, where the relative biomass values fall above the RY_A_ = RY_B_ diagonal line indicate that the growth of *Poa pratensis* was inhibited (RY_A_ < RY_B_). From years 2–6, the relative yields of *Poa + Elymus* and *Poa + Puccinellia* mixtures fall within the range of RTA < 1.0 and RTB > 1.0. This suggests that in these two mixed grasslands, interspecific competition between *Poa pratensis* and *Elymus sibiricus* was stronger than intraspecific competition (RYA < 1.0), whereas interspecific competition between *Elymus sibiricus* and *Puccinellia tenuiflora* was weaker than their intraspecific competition (RYB > 1.0). However, from years 3–6, the relative yield of the *Poa + Festuca* mixture falls within the range of RTA < 1.0 and RTB < 1.0, indicating that interspecific competition between *Poa pratensis* and *Festuca sinensis* exceeded intraspecific competition for both species (Fig 4A). The RYT of *Poa + Elymus* mixture was significantly greater than 1 in the second and third years (*P* < 0.05), but dropped below 1 in the fourth year (*P* < 0.05). This suggests that no intense competition existed between *Poa pratensis* and *Elymus sibiricus* during the second and third years; however, strong competition emerged from the fourth to the sixth years.

The RYT of *Poa + Festuca* mixture was significantly greater than 1 in the second year (*P* < 0.05); but significantly lower than 1 from the third to the sixth year (*P* < 0.05). This suggests that there was no intense competition between *Poa pratensis* and *Festuca sinensis* in the second year, but competition intensified from the third to the sixth years.

In contrast, the RYT of *Poa + Puccinellia* mixture was significantly lower than 1 in the second year (P < 0.05), but increased to values significantly greater than 1 from the third to sixth years (P < 0.05), suggesting that intense competition existed between *Poa pratensis* and *Puccinellia tenuiflora* in the second year, which subsided in later years (Fig 4B, S3 Table in S1 File).

### 3.3. Comprehensive evaluation of the production performance of cultivated grasslands

**Fig 5:**
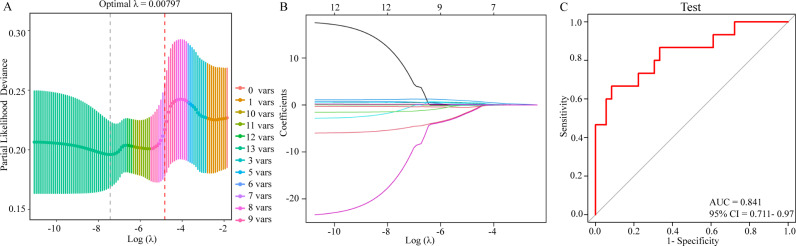
LASSO regression analysis and model evaluation of production performance in cultivated grasslands under mixed-sown and monoculture treatments. (A) LASSO Regression Model Convergence Results. (B) LASSO Regression Output. (C) ROC Model Prediction.

The optimal tuning parameter (λ) was determined through 10-fold cross-validation, which identified the ideal number of variables for the model. The λ value corresponding to the maximum within one standard error of the minimum mean squared error was found to be 1.79 × 10 ⁻ ^5^ (Fig 5A). As λ increased, the regression coefficients of the 12 independent variables gradually decreased, with some coefficients eventually shrinking to zero, effectively eliminating them from the model (Fig 5B). At λ = 1.79 × 10 ⁻ ^5^, eight variables were retained: CP, DMI, NDF, CF, stem-to-leaf ratio, leaf biomass, stem biomass, and forage yield. These selected variables and their corresponding coefficients were then used to develop a predictive model, which was subsequently applied to different treatment groups.

To assess the performance of the predictive model, receiver operating characteristic (ROC) analysis was conducted (Fig 5C). ROC analysis is a widely used statistical method for evaluating a model’s classification performance by plotting the true positive rate (sensitivity) against the false positive rate (1-specificity) across different threshold levels. The area under the ROC curve (AUC) quantifies the model’s discriminative power, with values closer to 1.0 indicating higher predictive accuracy. In this study, ROC analysis confirmed that the model effectively distinguished between mixed-sown and monoculture treatments in cultivated grasslands, achieving an AUC of 0.841 (95% CI: 0.711–0.97), demonstrating its strong predictive capability.

**Fig 6:**
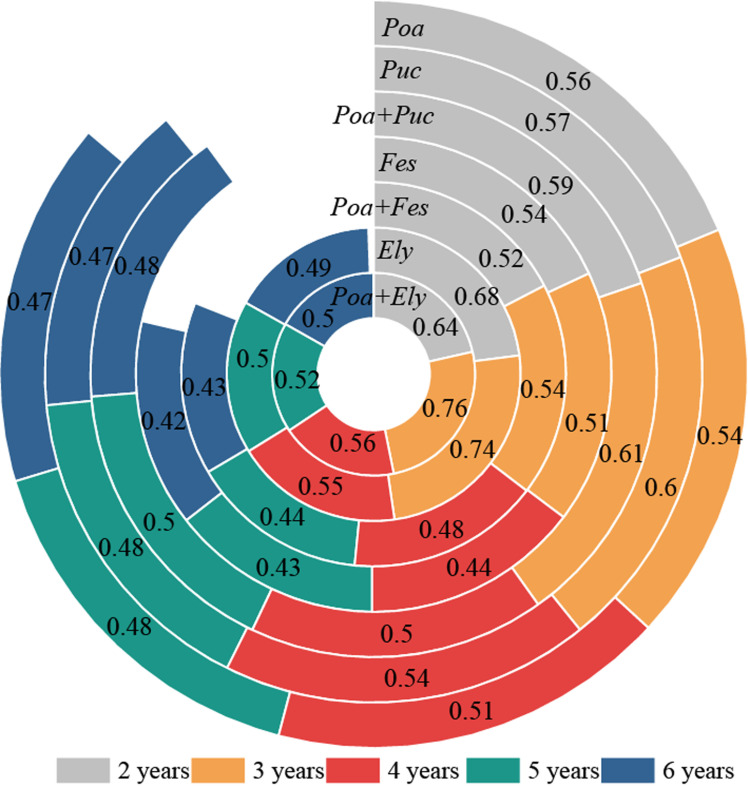
Comprehensive evaluation of cultivated grassland production performance.

The CRITIC method was employed to determine the weights of key indicators for evaluating the production performance of cultivated grasslands, including CP, DMI, NDF, CF, stem-to-leaf ratio, leaf biomass, stem biomass, and forage yield (Table 1). These weighted indicators were then used to calculate a comprehensive performance evaluation index using Equation 1 (Fig 6A, S4 Table in S1 File). The results indicate that the comprehensive performance index for *Poa* + *Elymus* and *Poa* + *Puccinellia* mixtures was higher than that of the monoculture grasslands. Conversely, the *Poa + Festuca* mixture exhibited a lower comprehensive performance index compared to the monoculture grasslands.

**Table 1:** Weighting of cultivated grassland production performance indicators using the CRITIC method.

Evaluation Indicators	Variability of indicators	Conflicting indicators	Amount of information	Weighting factor (%)
Crude protein (CP)	0.348	5.823	2.028	16.694
Neutral detergent fiber (NDF)	0.259	9.774	2.535	20.871
Crude fiber (CF)	0.21	7.589	1.595	13.129
Dry matter intake (DMI)	0.248	6.374	1.580	13.006
Yield	0.247	4.378	1.080	8.892
Stem biomass	0.230	4.447	1.021	8.403
Leaf biomass	0.198	4.638	0.919	7.563
Leaf/Stem ratio	0.261	5.318	1.390	11.443

## 4. Discussion

### 4.1. Impact of different cultivated grasslands on grassland production

Forage yield, a critical indicator of cultivated grassland productivity, was highest in the *Poa pratensis* + *Elymus sibiricus* mixture throughout the experimental period. However, when it came to yield stability, the *Poa pratensis* + *Puccinellia tenuiflora* mixture showed the best performance, achieving the highest stability value of 3.12. These findings suggest that the *Poa pratensis* + *Elymus sibiricus* mixture effectively addresses the low productivity issue often seen in monoculture *Poa pratensis* grasslands, while the *Poa pratensis* + *Puccinellia tenuiflora* mixture excels in yield stability. Therefore, the combination of *Poa pratensis* and *Puccinellia tenuiflora* exhibited superior performance in achieving long-term yield stability and high productivity in cultivated grasslands.

Forage nutritional quality is a crucial factor in evaluating the performance of mixed-sown grasslands. The stem-to-leaf ratio serves as a reliable indicator of forage palatability [17,18]. Among the analyzed mixtures, the combination of *Poa pratensis* and *Puccinellia tenuiflora* exhibited the lowest stem-to-leaf ratios in the second, third, fifth, and sixth years, indicating superior palatability.

Crude protein content is a key measure of forage nutritional value, while crude fiber, neutral detergent fiber (NDF), and acid detergent fiber (ADF) content are strong indicators of digestibility. The *Festuca sinensis* + *Poa pratensis* and *Puccinellia tenuiflora* + *Poa pratensis* mixtures demonstrated the highest crude protein content among all mixtures. Moreover, the study found that all mixed-sown combinations involving *Poa pratensis* resulted in higher crude protein content compared to monocultures. Notably, the *Puccinellia tenuiflora* and *Poa pratensis* mixture exhibited the lowest crude fiber content, further enhancing its nutritional value.

### 4.2. Impact of different cultivated grasslands on interspecific relationships

Plants in the same environment inevitably compete with varying intensity. This competition occurs not only between different species (interspecific competition) but also among individuals of the same species (intraspecific competition) [19]. Differences in the ability of plants to acquire and utilize resources lead to varying competitive abilities. The more similar the ecological requirements of plants, the more intense the competition [20,21]. Differences in a plant’s resource acquisition and utilization capabilities lead to varying levels of competitiveness. The more similar the ecological requirements of plants, the more intense the competition (Koffel et al., 2021; Pastore et al., 2021).

This study shows that *Poa pratensis* experiences growth inhibition when mixed with other forage grasses, as evidenced by the relative yield of competing species (RY_B_) surpassing that of *Poa pratensis* (RY_A_). This inhibition is mainly due to its vigorous root system, which strengthens belowground competition but limits aboveground competitiveness (CR < 1.0). *Poa pratensis* monoculture grasslands in Qinghai have exhibit lower aboveground biomass but accumulate more belowground biomass (Palit et al., 2021). During the mid-successional stage, these grasslands achieve higher community coverage and plant height, resulting in a gradual increase in both aboveground and belowground biomass. These findings align with the results of this study.

In the mixed-sown grassland of *Elymus sibiricus* and *Poa pratensis*, the relative total production (RYT) exceeded 1.0 in the second and third years. However, with grassland maturation, the RYT gradually decreased, becoming significantly lower than 1.0 in the four years. This suggests that niche differentiation occurred in the early years, reducing competition, due to the substantial height difference between the two species, which minimized light competition [22]. Furthermore, in the early stages of grassland establishment, underdeveloped root systems resulted in lower competition for soil nutrients, thereby reducing interspecific competition [23]. As the grassland aged, competition intensified due to the increasing root biomass of both species, intensifying competition for soil nutrients. Additionally, *Elymus sibiricus* exhibited signs of degradation over time, with a decline in plant height, exacerbating light competition between the two species. This increased competition contributed to the overall decline in the relative yield of the mixed-sown grassland [24]. In a mixed-sown grassland of *Festuca sinensis* and Kentucky bluegrass (*Poa pratensis*), the relative total yield (RYT) exceeded 1.0 in the second year, indicating minimal competition. However, as the grassland matured, the RYT significantly declined from the third to the sixth year, suggesting increasing competition for resources [25]. Relative biomass analysis (RY) revealed that both *Festuca sinensis* and *Poa pratensis* had values below 1.0 during the third and fourth years, indicating mutual inhibition. These findings suggest that interspecific competition intensified over time, making the mixed-sown approach for these two species less effective [26].In a mixed-sown grassland of *Puccinellia tenuiflora* and *Poa pratensis*, the relative total yield (RYT) was initially less than 1.0 in the second year, indicating intense competition for resources. However, as the grassland matured, the RYT significantly from the third to the sixth year, surpassing 1.0. This suggests that early competition between the two species gradually subsided over time. The interspecific relationship between *Puccinellia tenuiflora* and *Poa pratensis* in a mixed-sown system demonstrates a high level of compatibility. This observed trend suggests a mutualistic relationship, with both species benefiting from coexistence. These findings indicate that combining *Puccinellia tenuiflora* and *Poa pratensis* in a mixed-sown system can effectively reduce competition and enhance overall productivity, ultimately leading to greater yield stability and improved forage yield [27].

### 4.3. Production performance and stability assessment of mixed-sown and monoculture grasslands

Effectively assessing the production performance and interspecific relationships of different grasslands is a key aspect of forage species evaluation [28]. Due to the considerable variation in forage yield and nutritional quality among different grasslands, accurately evaluating them using only a single or a few indicators presents significant challenges [29]. To address this, the present study applied LASSO regression and ROC curve analysis to identify key indicators of production performance across various grassland treatments. The results indicate that crude protein (CP), dry matter intake (DMI), neutral detergent fiber (NDF), crude fiber (CF), stem-to-leaf ratio, leaf biomass, stem biomass, and forage yield were effective predictors of both mixed-sown and monoculture grasslands (AUC = 0.841, 95% CI: 0.711–0.97).

Furthermore, the CRITIC method was employed to assign weights to these indicators, enabling the calculation of a comprehensive production performance score for different grassland types. The findings revealed a declining trend in production performance with increasing cultivation duration. Among the evaluated treatments, the mixed-sown grassland of *Poa pratensis* and *Elymus sibiricus* demonstrated the highest overall production performance. To address the challenge of insufficient productivity in *Poa pratensis* grasslands, incorporating *Elymus sibiricus* into a mixed-sowing strategy is recommended as an effective approach to enhance forage production and sustainability.

Although the mixture of *Elymus sibiricus* and *Poa pratensis* exhibited superior forage nutritional value and total yield, the mixture of *Puccinellia tenuiflora* and *Poa pratensis* demonstrated the highest yield stability, significantly outperforming the other treatments. Throughout the study period, the relative total yield (RTY) of the *Puccinellia tenuiflora* and *Poa pratensis* mixture consistently exceeded 1.0, indicating enhanced resource complementarity and reduced interspecific competition over time. This finding suggests that the *Puccinellia tenuiflora* and *Poa pratensis* mixture possesses a greater capacity to adapt to environmental fluctuations, ensuring more stable productivity in the long term [30]. In contrast, the mixtures of *Elymus sibiricus* and *Poa pratensis* and *Festuca sinensis* and *Poa pratensis* exhibited a declining trend in RYT values, with significantly lower yield stability compared to the *Puccinellia tenuiflora* and *Poa pratensis* mixture. These results indicate that interspecific competition within these mixtures led to inefficient resource utilization, ultimately limiting their long-term productivity. Overall, the findings suggest that the combination of *Puccinellia tenuiflora* and *Poa pratensis* is more suitable for establishing stable and sustainable mixed-sown grasslands. Given its optimal balance between yield performance and resilience to environmental variability, the *Puccinellia tenuiflora* and *Poa pratensis* mixture is recommended for long-term and sustainable grassland utilization.

## 5. Conclusions

This study provides a comprehensive evaluation of the production performance and interspecific relationships of various mixed-sown and monoculture grasslands on the Qinghai-Tibetan Plateau. The results indicate that the mixed-sown grassland of *Elymus sibiricus* and *Poa pratensis* significantly improves overall production performance, effectively addressing the low productivity issues observed in monoculture *Poa pratensis* systems. However, in terms of yield stability and relative total yield (RTY), the mixture of *Puccinellia tenuiflora* and *Poa pratensis* demonstrated the highest stability and resource complementarity, outperforming other treatments in sustaining long-term productivity. The RTY of this mixture consistently exceeded 1.0 from the third to the sixth year, reflecting a gradual improvement in resource utilization efficiency and a reduction in interspecific competition over time. In contrast, the *Elymus sibiricus* + *Poa pratensis* and *Festuca sinensis* + *Poa pratensis* mixtures exhibited declining RTY values and lower yield stability, indicating stronger interspecific competition and less efficient resource utilization. Therefore, for the long-term and sustainable utilization of cultivated grasslands, the combination of *Poa pratensis* and *Puccinellia tenuiflora* is recommended as the optimal strategy to achieve both yield stability and high productivity.

## Supporting information

S1 File The manuscript data comprises the original data from this experiment, where Table S1 contains the raw data for Fig 1; Table S2 includes the raw data for Figs 2 and 3; Table S3 provides the raw data for Fig 4; and Table S4 presents the raw data for Fig 6. (DOCX)
