# Supplementary material for: Production performance in cultivated mixed-sown grasslands combining Poa pratensis L. and various Poaceae forage grasses
Source: PLoS One. 2025 May 19;20(5):e0324084. doi: 10.1371/journal.pone.0324084 (PMC12088026; doi:10.1371/journal.pone.0324084)
Supplement: S1 File — (DOCX) [file pone.0324084.s001.docx]

Table S1 Forage yield of different cultivated grasslands

|  | Yield stability | 2 years Yeld | 3 years Yeld | 4 years Yeld | 5 years Yeld | 6 years Yeld |
| --- | --- | --- | --- | --- | --- | --- |
| *Poa* | 2.63±0.21B | 91.65±26.5AB | 120.91±9.64A | 57.28±5.61B | 49.67±5.46B | 50.11±4.43B |
| *Ely* | 2.39±0.22B | 411.3±47.92B | 511.78±54.88A | 254.27±22.9C | 197.47±10.7C | 176.63±9.79D |
| *Fes* | 2.51±0.14B | 137.52±16.54A | 159.66±20.79A | 155.62±16.06A | 64.69±7.29B | 54.28±5.29B |
| *Puc* | 2.49±0.13B | 90.84±15.28B | 151.31±9.99A | 77.84±7.3B | 58.05±6.31C | 55.36±5.85C |
| *Poa+Ely* | 2.12±0.17CD | 358.42±32.31B | 511.51±56.32A | 200.48±22.36C | 188.35±9.83C | 158.79±9.6C |
| *Poa+Fes* | 1.83±0.12D | 158.31±15.44A | 77.69±7.77B | 49.02±5.41C | 52.06±5.03C | 48.43±4.65C |
| *Poa+Puc* | 3.12±0.11A | 140.81±14.71AB | 161.8±18.21A | 123.73±13.36B | 74.96±6.03C | 68.63±6.62C |

Note: Different letters in the figure indicate significant differences in forage yield among grasslands across different planting years (*P* < 0.05).

Table S2 Nutritional quality of different cultivated grasslands

| Treatment | Stem/Leaf ratio | Crude protein (CP) mg/g | Neutral detergent fiber (NDF) % | Acid detergent fiber (ADF) % | Crude fiber (CF) % | Digestible dry matter (DDM) | Dry matter intake (DMI) | Relative feed value (RFV) |
| --- | --- | --- | --- | --- | --- | --- | --- | --- |
| 2 years *Poa* | 1.84±0.04Be | 18.85±0.77Aa | 33.97±1.25Cc | 35.64±1.96Aa | 41.38±2.33Ba | 61.15±1.53Bd | 3.55±0.14Aa | 167.85±10.39Aa |
| 2 years *Elymus* | 4.29±0.05Aa | 14.54±0.61Ab | 44.44±1.93Bab | 26.75±1.27ABcd | 33.98±1.74Bbc | 68.08±0.99ABab | 2.72±0.13Ab | 142.91±8.48ABb |
| 2 years *Poa+Elymus* | 3.17±0.12Bc | 16.09±0.94Ab | 40.72±2.22Bb | 26.19±1.27NSd | 29.02±1.52Cd | 68.51±0.99NSa | 2.97±0.18Ab | 157.16±11.2Aab |
| 2 years *Festuca* | 3.39±0.17Ab | 16.07±0.92Bb | 33.68±1.68Cc | 30.83±1.43NSb | 38.95±1.89NSa | 64.89±1.11NSb | 3.58±0.19Aa | 179.92±12.39Aa |
| 2 years *Poa+Festuca* | 3.36±0.12Bbc | 18.22±0.86Aa | 35.99±2.21Bc | 30.32±1.58Bbc | 33.35±1.84Bc | 65.3±1.23Abc | 3.36±0.22Aa | 169.6±14.07Aa |
| 2 years *Puccinellia* | 3.59±0.12Ab | 12.72±0.64Ac | 36.24±1.53Cc | 29.73±1.84Abcd | 37.66±1.43Aab | 65.76±1.44Babc | 3.33±0.15Aa | 169.28±10.99Aa |
| 2 years *Poa+Puccinellia* | 2.31±0.09Ad | 14.82±0.58Bb | 45.38±1.89Ba | 27.98±1.7ABbcd | 28.42±1.79NSd | 67.12±1.33ABabc | 2.66±0.12Ab | 137.97±8.58Ab |
| 3 years *Poa* | 2.39±0.07Ad | 18.52±0.98Aa | 36.39±2.16Cb | 32.18±2.13ABa | 29.19±1.29Cc | 63.85±1.66ABb | 3.32±0.21Aa | 164.07±14.11Aa |
| 3 years *Elymus* | 2.93±0.07Dc | 15.28±0.81Acd | 46.67±2.36ABa | 23.94±1.48Bb | 37.92±1.86Aa | 70.27±1.16Aa | 2.59±0.14ABb | 140.55±9.56ABab |
| 3 years *Poa+Elymus* | 3.97±0.23Ab | 16.42±0.69Abc | 43.45±2.13Ba | 24.51±1.24NSb | 36.47±1.55Ba | 69.82±0.97NSa | 2.78±0.15Ab | 149.99±9.71Aab |
| 3 years *Festuca* | 3.51±0.19Ab | 18.02±0.91Aab | 42.32±2.24Ba | 29.68±1.22NSa | 31.57±1.49NSbc | 65.79±0.95NSb | 2.85±0.16Bb | 145.18±9.95Bab |
| 3 years *Poa+Festuca* | 4.84±0.17Aa | 18.89±0.73Aa | 41.27±2.52Bab | 29.07±1.81Ba | 35.04±1.98Bab | 66.27±1.41Ab | 2.93±0.2Bb | 150.16±12.65Aab |
| 3 years *Puccinellia* | 2.29±0.12Bd | 13.88±0.75Ad | 42.56±1.67Ba | 29.45±1.82Aa | 31.76±1.5Bbc | 65.97±1.42Bb | 2.83±0.12Bb | 144.57±8.91Bab |
| 3 years *Poa+Puccinellia* | 1.51±0.11Ce | 18.96±0.81Aa | 45.38±2.88Ba | 30.49±1.96Aa | 31.39±1.87NSbc | 65.17±1.53Bb | 2.67±0.18Ab | 134.34±11.87Ab |
| 4 years *Poa* | 2.07±0.09Bcd | 9.18±0.53Ba | 65.21±2.87Aa | 32.19±2.24ABab | 46.82±2.16Ab | 63.83±1.75ABbc | 1.85±0.09Bb | 91.35±6.64Bb |
| 4 years *Elymus* | 3.72±0.23Ba | 6.89±0.39Bc | 51.16±2.71Ab | 27.36±0.98Ac | 29.31±1.55Cd | 67.59±0.76Ba | 2.36±0.14Ba | 123.34±7.84Ba |
| 4 years *Poa+Elymus* | 3.3±0.12Bb | 7.81±0.49Bbc | 51.01±3.26Ab | 27.33±1.65NSc | 42.2±2.02Ac | 67.63±1.29NSa | 2.37±0.17Ba | 124.02±10.7Ba |
| 4 years *Festuca* | 3.23±0.14Bb | 7.49±0.42Cbc | 61.77±3.12Aa | 31.65±1.81NSab | 41.21±1.76NSc | 64.26±1.41NSbc | 1.96±0.11Cb | 97.14±7.23Cb |
| 4 years *Poa+Festuca* | 1.93±0.07Cd | 7.77±0.38Bbc | 59.27±3.75Ba | 34.86±1.64Aa | 56.65±3.09Aa | 61.76±1.28Bc | 2.04±0.14Cb | 97.42±7.84Bb |
| 4 years *Puccinellia* | 1.68±0.11De | 7.06±0.4Bc | 58.99±2.74Aa | 26.54±1.74ABc | 28.25±1.71Cd | 68.24±1.36ABa | 2.05±0.11Cb | 107.94±7.14Cab |
| 4 years *Poa+Puccinellia* | 2.23±0.12Ac | 8.06±0.33Cb | 58.29±3.91Aa | 28.69±1.22ABbc | 31.22±1.89NSd | 66.57±0.95ABab | 2.08±0.15Bb | 106.85±9.02Bab |
| 5 years *Poa* | 1.97±0.07Bd | 9.09±0.62Ba | 59.72±3.59ABab | 29.07±1.89Ba | 42.09±2.37ABb | 66.27±1.48Ab | 2.03±0.13Bcd | 103.75±8.54Bcd |
| 5 years *Elymus* | 3.59±0.22BCa | 7.06±0.35Bb | 46.72±2.07ABd | 24.75±1.63ABb | 28.81±1.26Cc | 69.64±1.27ABa | 2.58±0.13Aa | 139.03±8.46Aa |
| 5 years *Poa+Elymus* | 3.14±0.15Ba | 7.44±0.41Bb | 51.33±2.04Acd | 27.36±1.5NSab | 39.58±2.07ABb | 67.61±1.17NSab | 2.35±0.1Bb | 122.79±6.79Bab |
| 5 years *Festuca* | 2.57±0.14Cb | 6.69±0.42Cb | 62.19±2.67Aa | 30.28±1.56NSa | 38.17±2.14NSb | 65.33±1.21NSb | 1.94±0.09Cd | 97.99±6.11Cd |
| 5 years *Poa+Festuca* | 2.05±0.11Cc | 7.31±0.39Bb | 60.67±2.97Aab | 30.92±1.99ABa | 51.74±2Aa | 64.83±1.55ABb | 1.99±0.11Ccd | 99.77±7.42Bd |
| 5 years *Puccinellia* | 1.89±0.12Cd | 7.05±0.35Bb | 55.09±2.56Abc | 24.28±1.6Bb | 25.86±1.67CDc | 70±1.25Aa | 2.19±0.11Cbc | 118.56±7.66Cbc |
| 5 years *Poa+Puccinellia* | 1.83±0.09Bd | 7.45±0.36Cb | 56.88±2.84Aabc | 27.11±1.77ABab | 29.74±1.76NSc | 67.79±1.38ABab | 2.13±0.12Bbcd | 111.29±7.98Bbcd |
| 6 years *Poa* | 1.57±0.17Cd | 8.92±0.32Ba | 58.18±3.2Bab | 28.6±1.25Bab | 42.06±2.26ABb | 66.64±0.98Ade | 2.08±0.13Bcd | 106.99±7.7Bcd |
| 6 years *Elymus* | 3.46±0.21Ca | 6.85±0.39Bc | 43.28±2.27Bd | 24.68±1.31ABde | 27.33±1.37Cd | 69.69±1.02ABab | 2.79±0.16ABa | 150.36±10.34ABa |
| 6 years *Poa+Elymus* | 3.23±0.24Ba | 6.83±0.38Bc | 49.08±2.71Acd | 27.54±1.06NSbcd | 36.29±1.31Bc | 67.46±0.83NSbcd | 2.46±0.15Bb | 128.35±8.44Bb |
| 6 years *Festuca* | 1.93±0.12Db | 6.97±0.27Cc | 62.54±2.68Aa | 28.35±1.62NSabc | 38.47±1.66NSbc | 66.83±1.27NScde | 1.93±0.09Cd | 99.68±6.21Cd |
| 6 years *Poa+Festuca* | 1.74±0.09Dc | 7.03±0.37Bc | 57.39±3.53Aab | 30.99±1.92ABa | 54.09±3.39Aa | 64.78±1.49ABe | 2.11±0.14Ccd | 105.54±8.96Bcd |
| 6 years *Puccinellia* | 1.97±0.07Cb | 6.96±0.39Bc | 55.29±2.75Abc | 24.28±1.7Be | 23.29±1.39Dd | 70.75±0.81Aa | 2.19±0.12Ccd | 119.43±7.51Cbc |
| 6 years *Poa+Puccinellia* | 1.71±0.15Bc | 7.98±0.42Cb | 54.26±2.42Abc | 25.25±1.69Bcde | 27.43±1.42NSd | 69.25±1.32Aabc | 2.23±0.11Bbc | 119.07±7.77ABbc |

Note: Different letters in the figure indicate significant differences in forage yield among grasslands across different planting years (*P* < 0.05). Figure 1A illustrates the forage yield of *Poa + Elymus* mixtures, *Poa* monoculture, and *Elymus* monoculture. Figure 1B shows the forage yield of *Poa + Festuca* mixtures, *Poa* monoculture, and *Festuca* monoculture. Figure 1C presents the forage yield of *Poa + Puccinellia* mixtures, *Poa* monoculture, and *Puccinellia* monoculture. Figure 1D depicts the yield stability of different grasslands.

Table S3 Interspecific Relationships in Different Mixed Grasslands

| Treatment | RY_A_ | RY_B_ | RYT |
| --- | --- | --- | --- |
| 2 years *Poa+Elymus* | 0.91 | 1.47 | 1.19±0.02 * |
| 2 years *Poa+Festuca* | 0.59 | 1.88 | 1.23±0.03 * |
| 2 years *Poa+Puccinellia* | 0.4 | 1.3 | 0.85±0.02 * |
| 3 years *Poa+Elymus* | 0.39 | 1.95 | 1.17±0.06 * |
| 3 years *Poa+Festuca* | 0.39 | 0.79 | 0.58±0.01 * |
| 3 years *Poa+Puccinellia* | 0.51 | 1.87 | 1.19±0.01 * |
| 4 years *Poa+Elymus* | 0.3 | 1.52 | 0.91±0.02 * |
| 4 years *Poa+Festuca* | 0.58 | 0.6 | 0.36±0.00 * |
| 4 years *Poa+Puccinellia* | 0.35 | 2.19 | 1.27±0.02 * |
| 5 years *Poa+Elymus* | 0.29 | 1.78 | 1.03±0.05 ns |
| 5 years *Poa+Festuca* | 0.66 | 0.66 | 0.89±0.03 * |
| 5 years *Poa+Puccinellia* | 0.33 | 2.26 | 1.3±0.02 * |
| 6 years *Poa+Elymus* | 0.24 | 1.83 | 1.03±0.05 ns |
| 6 years *Poa+Festuca* | 0.72 | 0.79 | 0.95±0.02 * |
| 6 years *Poa+Puccinellia* | 0.29 | 2.2 | 1.24±0.02 * |

Note: "*" indicates a significant difference in RYT from 1 (P < 0.05).

Table S4 Comprehensive Evaluation of Cultivated Grassland Production Performance

|  | 2 years Yeld | 3 years Yeld | 4 years Yeld | 5 years Yeld | 6 years Yeld |
| --- | --- | --- | --- | --- | --- |
| *Poa* | 0.55646 | 0.54194 | 0.51274 | 0.485 | 0.47138 |
| *Ely* | 0.68408 | 0.73776 | 0.55009 | 0.50343 | 0.48549 |
| *Fes* | 0.53704 | 0.51379 | 0.44176 | 0.42681 | 0.42157 |
| *Puc* | 0.57195 | 0.59545 | 0.54079 | 0.48074 | 0.46572 |
| *Poa+Ely* | 0.63835 | 0.75579 | 0.56051 | 0.52374 | 0.49999 |
| *Poa+Fes* | 0.51738 | 0.5359 | 0.48317 | 0.44413 | 0.43361 |
| *Poa+Puc* | 0.58905 | 0.60625 | 0.5038 | 0.4969 | 0.48424 |
